# Assessment of EAPSDAS in critically ill patients with antiphospholipid syndrome

**DOI:** 10.1016/j.ero.2026.100225

**Published:** 2026-08-18

**Authors:** Lévi-Dan Azoulay, Alexis Mathian, Zahir Amoura, Marc Pineton de Chambrun

**Affiliations:** 1 Sorbonne Université, Laboratoire d’Imagerie Biomédicale, Inserm, CNRS, Paris, France; 2 Sorbonne Université, Assistance Publique-Hôpitaux de Paris, Service de Médecine Interne 2, Centre National de Référence du Lupus, Hôpital Pitié-Salpêtrière, Paris, France; 3 Sorbonne Université, Assistance Publique-Hôpitaux de Paris, Service de Réanimation Médicale, Paris, France

Dear Editor,

The European Alliance of Associations for Rheumatology (EULAR) antiphospholipid syndrome (APS) disease activity score (EAPSDAS) was recently coined as a novel tool to measure disease activity in patients with APS [1]. Importantly, some patients with an active APS may require intensive care unit (ICU) admission because of organ dysfunctions caused by macrovascular and/or microvascular thromboses with or without meeting the catastrophic APS (CAPS) classification criteria [2]. To date, whether fulfilment of these criteria is associated with a higher EAPSDAS score is unknown. In addition, mortality ranges between 20% and 50% in this setting, but it is unknown whether the EAPSDAS score could have prognostic value in predicting patients’ outcomes [3].

This study aimed to explore the relationship between the EAPSDAS score and both CAPS classification criteria and in-hospital mortality in critically ill thrombotic patients with APS.

This French, national, multicentre, retrospective study, conducted from January 2000 to September 2018, included all patients with APS admitted to 24 participating centres’ ICUs with any new thrombotic (arterial, venous, or microvascular) manifestation, as previously published [3]. Data from this cohort have been described previously [3,4]. APS was defined using the 2006 APS international classification criteria [5].The EAPSDAS score was calculated for each patient at ICU admission [1]. CAPS classification criteria were assessed, and patients were classified into 3 subgroups: no CAPS, probable CAPS classification criteria, and definite CAPS classification criteria. Triple therapy was defined by anticoagulation, corticosteroid, and intravenous immunoglobulin or plasma exchange (IVIG/PLEX) therapies. The primary outcome was in-hospital mortality. Approval of the Ethics Committee of the French Intensive Care Society (#CE SRLF17-30) was obtained.

Statistical analyses were performed using R (R Core Team, version 4.2.3). First, the distribution of the EAPSDAS scores in the cohort was assessed and divided into quartiles. Second, the risk of in-hospital mortality was compared across the quartile-based subgroups using Kaplan-Meier survival curves and Log-rank tests.

A total of 134 critically ill patients with APS were included (aged 46 ± 15 years, 70% female). Main manifestations included arterial thrombosis in 58 patients (43%), venous thrombosis in 65 (49%), intra-alveolar haemorrhage in 6 (4%), adrenal haemorrhage in 12 (9%), and livedo reticularis in 18 (13%). Mean platelet count was 99 ± 84 G/L and was < 50 lt; 50 G/L in 37 patients (27%) at admission. CAPS classification criteria were as follows: no CAPS classification criteria in 68 patients (51%), probable CAPS classification criteria in 56 (42%), and definite CAPS classification criteria in 10 (7%). Mean ± SD and median (IQR) of the EAPSDAS scores were 171 ± 80 and 157 (IQR, 134-224), respectively (Figure 1A). Overall, no significant association was found between CAPS criteria and EAPSDAS scores (no CAPS classification criteria: 161 ± 75; probable CAPS: 187 ± 85 and definite CAPS: 160 ± 42; *P* = .2). A total of 128 patients (96%) received anticoagulation therapy, and 78 (58%) received triple therapy. Overall, the in-hospital death rate was 23%, but no association was found with the EAPSDAS score quartiles on survival analysis (log-rank *P* = .7, Fig, B). In addition, the EAPSDAS score did not differ according to in-hospital mortality status (in-hospital death: 176 ± 68 vs discharged alive: 171 ± 82, *P* = .7)

**Figure 1:**
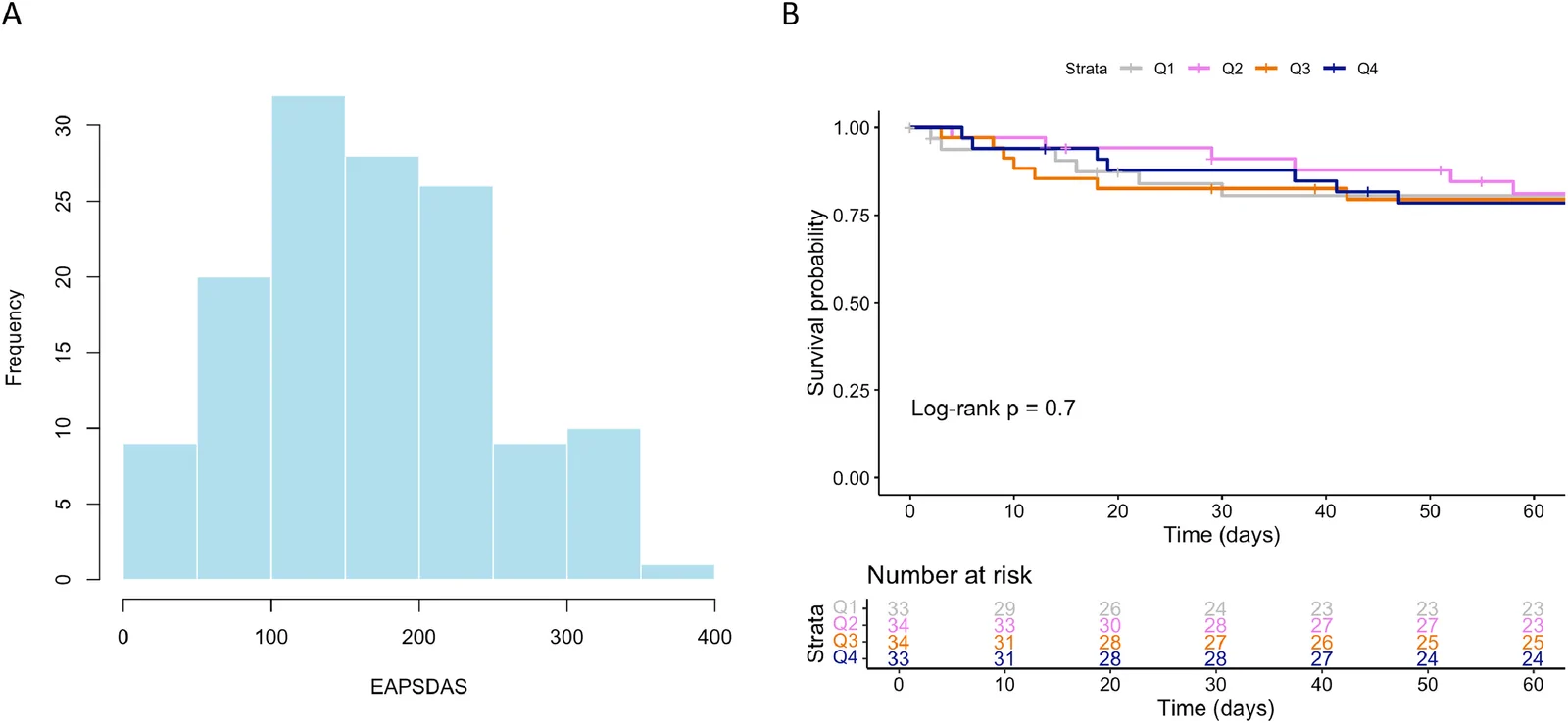
Distribution and prognostic value of EAPSDAS in a cohort of critically ill patients with antiphospholipid syndrome. (A) Distribution of EAPSDAS in the cohort. (B) Kaplan-Meier survival curve of in-hospital mortality. EAPSDAS values were divided into quartiles with cutoffs at 134, 157, and 224, yielding four groups of equal size (Q1 ≤134, Q2 135-157, Q3 158-224, Q4 ≥225). EAPSDAS, European Alliance of Associations for Rheumatology antiphospholipid syndrome disease activity score.

Overall, this study identified that EAPSDAS scores were markedly elevated but not associated with CAPS classification criteria.1 These findings highlighted the distinct value of these instruments, with the EAPSDAS score functioning as a disease activity score, whereas CAPS classification criteria serve primarily as a classification framework. In addition, the EAPSDAS score did not predict outcomes. This further reinforces prior studies that demonstrated that outcomes are best predicted by conventional organ dysfunction scores in critically ill patients with APS.3 These findings should be interpreted with caution, as components of the EAPSDAS score may be absent at ICU admission or confounded by concurrent pathophysiological processes in critically ill patients. In conclusion, the EAPSDAS score may retain a complementary role in the assessment of critically ill patients with APS.

## Competing interest

ZA reports a relationship with GSK, AstraZeneca, Roche, Novartis, Amgen, and Kezar that includes consulting or advisory and funding grants. All other authors declare that they have no known competing financial interests or personal relationships that could have appeared to influence the work reported in this paper. This work was performed without any relationship with the industry.
